# Hemophagocytic Lymphohistiocytosis: A Rare Cause of Pyrexia of Unknown Origin

**DOI:** 10.7759/cureus.23368

**Published:** 2022-03-21

**Authors:** Salman Khan, Fahad Naim, Hameed Ullah, Falak Niaz, Muhammad Bilal

**Affiliations:** 1 Internal Medicine, Khyber Teaching Hospital, Peshawar, PAK

**Keywords:** inflammation, immune activation, cytopenias, fever, hemophagocytic lymphohistiocytosis (hlh)

## Abstract

Hemophagocytic lymphohistiocytosis (HLH) is a life-threatening syndrome due to excessive immune activation leading to hyperinflammation. It may be familial due to mutations in immune regulatory genes, especially genetic defects of lymphocyte toxicity. The sporadic cases are triggered by infections (mostly viral), malignancies, and autoimmune diseases. Herewith we report the case of a 20-year-old male with febrile illness who was ultimately diagnosed with HLH.

## Introduction

Hemophagocytic lymphohistiocytosis (HLH) is an aggressive syndrome of excessive inflammation and tissue damage due to abnormal immune activation. The dysregulated immune state is caused by a lack of normal downregulation by activated macrophages and lymphocytes, leading to excess cytokine release resulting in multiorgan failure [1]. It most frequently affects infants from birth to 18 months of age, but the disease can also be observed in children and adults.

It can be familial or sporadic and can be triggered by a variety of events that disrupt immune homeostases like infections, malignancy, and autoimmune disorders. HLH usually has a poor outcome with median overall survival of only 7.6 months, and diagnosis is usually delayed due to its wide spectrum of clinical findings [2]. Herein we demonstrate a case of HLH who presented with prolonged fever and cytopenias.

## Case presentation

A 20-year-old male with no previous medical history presented to us with high-grade intermittent fever for the last three months. He also complained of anorexia, nausea, and unintentional weight loss. He consulted multiple general practitioners (GPs) for non-resolving fever, which was empirically treated with antibacterials, antimalarials, and antipyretics. During these visits, he was found to have pancytopenia but was not addressed. Dengue and malarial serology was negative.

On examination, he had pallor and splenomegaly (3 cm below the costal margin). His temperature was 101^o^ F, blood pressure was 100/60, respiratory rate of 16 per minute, heart rate of 120/min, and oxygen saturation of 99% at room air. The rest of the systemic examination was unremarkable.

Blood tests revealed normochromic normocytic anemia: Hb was 8.2, mean corpuscular volume (MCV) was 78, mean corpuscular hemoglobin concentration (MCHC) was 3, leukopenia (white blood cells [WBC] count was 1,600/cm^3^ with an absolute neutrophilic count of 1,088/cm^3^), severe thrombocytopenia (platelet count was 22,000/cm^3^), raised bilirubin (serum bilirubin was 1.39), raised alanine transaminase (ALT was 116 U/L), raised alkaline phosphatase (ALP was 260 U/L) and increased lactate dehydrogenase (LDH was 1,860 U/L). The rest of the baseline investigations were normal. Erythrocyte sedimentation rate (ESR) was high (60 mm/hr), and C-reactive protein was 103 mg/L. Serum ferritin was extremely elevated (10,350 ng/ml). Chest X-ray was normal, and abdominal ultrasonography revealed only splenomegaly with a spleen size of 18 cm. Peripheral smear showed anisocytosis, hypochromia, and red blood cells (RBC) fragmentation. Reticulocyte count was only 0.3%, due to which bone marrow biopsy was planned.

Bone marrow aspiration revealed cellular marrow with hemophagocytes and increased histiocytes. The high ferritin levels narrowed down our differential diagnosis to sepsis, juvenile idiopathic arthritis (JIA) with macrophage activation syndrome (MAS), and HLH. Based on the clinical features and laboratory findings, sepsis and JIA were ruled out. Further evaluation revealed hypertriglyceridemia (3.3 mmol/L), hypofibrinogenemia (127 mg/dL) and hyponatremia (130 mmol/L). He was diagnosed with hemophagocytic lymphohistiocytosis (HLH) based on HLH-2004 criteria [3] and HScore of 249.

**Figure 1:**
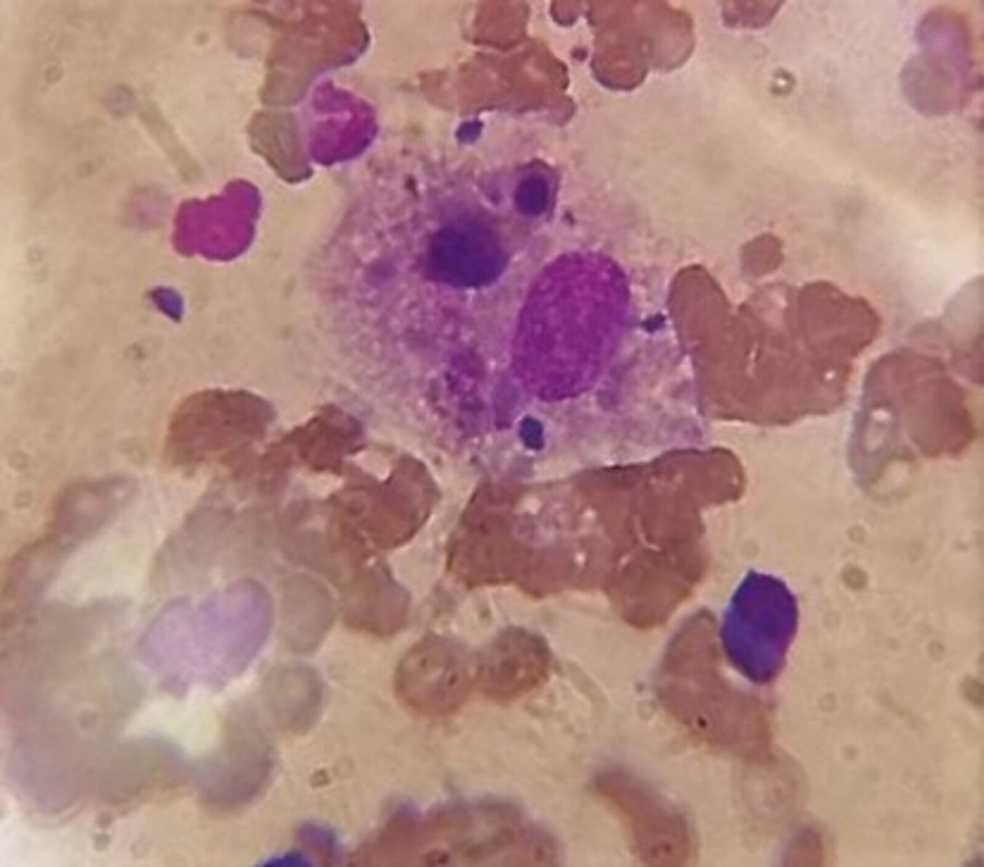
Bone marrow aspirate of the patient showing hemophagocytosis

In order to find out the triggering cause of HLH, we did hepatitis B surface (HBS) antigen, anti-hepatitis C virus (HCV) antibody, and anti-HIV antibody tests, which were negative. Other infectious diseases that are common in this region of the world, including malaria, dengue, SARS-CoV-2, and tuberculosis, were negative. Investigations for other viral illnesses, i.e., Epstein-Barr virus (EBV), cytomegalovirus (CMV), and parvovirus, couldn't be done due to lack of clinical features and non-availability of tests in the institute. Blood and urine cultures were negative. Cerebrospinal fluid (CSF) studies were not done because of the normal neurological status of the patient. Autoimmune profile (extractable nuclear antigen profile) came out to be negative. CT scan of thorax, abdomen, and pelvis with contrast revealed only splenomegaly with no evidence of malignancy.

**Figure 2:**
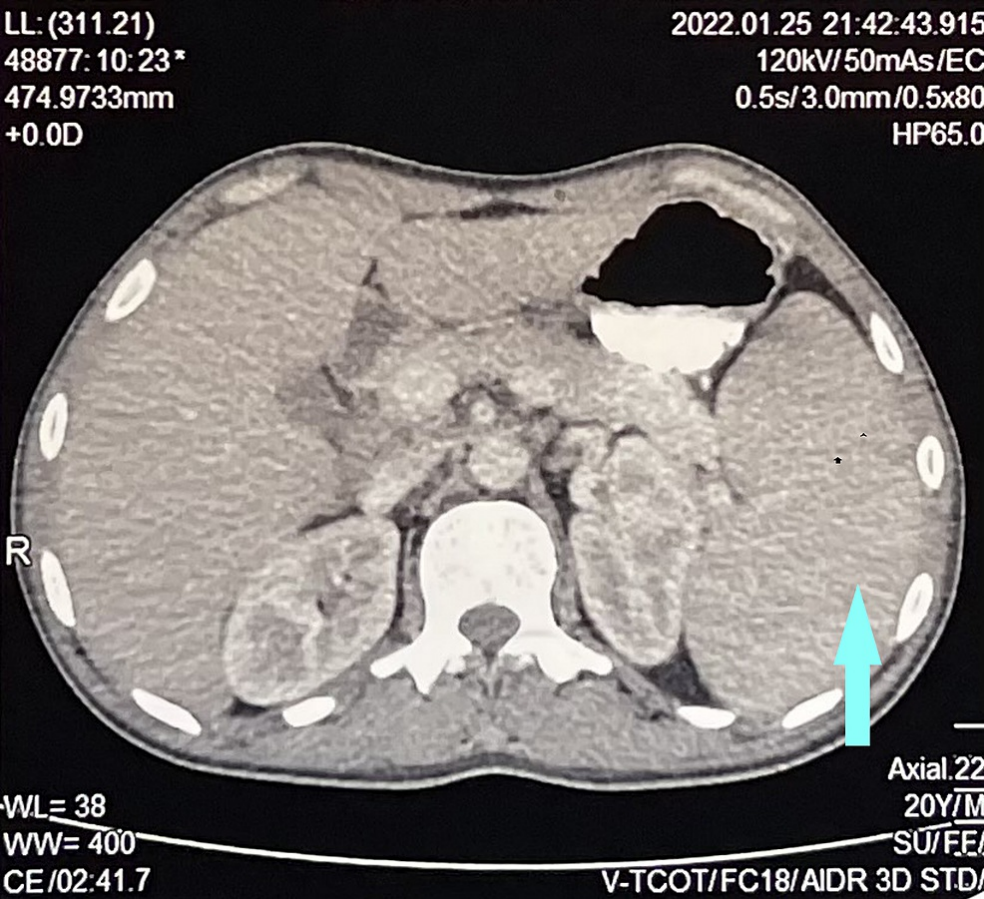
CT scan image of the patient's abdomen showing enlarged spleen

We couldn't proceed with immunologic and genetic testing due to non-availability and financial issues. We started the HLH-2004 protocol, and the patient showed immense improvement after just two weeks of therapy. His cell counts improved with a WBC of 2,800/cm^3^, a hemoglobin count of 9.2 g/dl, and a platelet count of 150,000/cm^3^. Ferritin levels were only 846 ng/ml (Table 1). He was advised for regular weekly follow-up.

**Table 1:** Clinical and laboratory parameters before and after treatment WBC - white blood cells; ALT - alanine transaminase

Parameter	Normal range	Before treatment	After two weeks of treatment
Temperature (°C)	36-37.2	38.5	37
Hemoglobin (g/dl)	11.5-17.5	8.2	9.2
WBC count (per cm^3^)	4k-11k	1.6k	2.8k
Platelet count (per cm^3^)	150k-450k	22k	150k
Serum ferritin (ng/ml)	30-400	10,350	846
Bilirubin (mg/dl)	0.1-1.0	1.39	0.74
ALT (U/L)	10-50	116	109
D-dimers (ng/ml)	<500	4491	2617

## Discussion

In HLH, natural killer cells and cytotoxic T lymphocytes fail to inhibit activated macrophages. This lack of homeostasis results in uncontrolled CD8+ T cell and macrophage activation with grossly elevated levels of different cytokines, which leads to the multiorgan damage observed in HLH [4,5]. Cytokines found at extremely high levels in the plasma of patients with HLH include interferon-gamma (IFN gamma), the chemokine CXCL9 (which is regulated by IFN gamma), tumor necrosis factor-alpha (TNF-alpha), interleukins (IL) such as IL-6, IL-10, IL-12 and the soluble IL-2 receptor [6,7].

Patients usually present with non-resolving fever and involvement of multiple organs, due to which they are often misdiagnosed. Fever, splenomegaly, and cytopenias are the most common manifestations of HLH [8]. Serum ferritin level can provide a clue as level >10,000 ng/ml is almost 90% sensitive and 96% specific for HLH [9]. Almost all the HLH patients have a deranged liver function, and liver enzyme levels greater than three times the normal limit has been reported in 50-90% of cases [10]. Other features include a deranged coagulation profile, hypertriglyceridemia, and hypofibrinogenemia. HLH can be diagnosed using the HLH-2004 criteria (Table 2) [3]. A scoring system named HScore has been developed to show the probability of HLH [11].

**Table 2:** HLH-2004 diagnostic criteria HLH - hemophagocytic lymphohistiocytosis; NK cells - natural killer cells; IL - interleukins

The diagnosis of HLH can be established if criterion 1 or 2 is fulfilled		
1	A molecular diagnosis consistent with HLH	
2	Diagnostic criteria for HLH fulfilled (5 of the 8 below)	
	a	Fever
	b	Splenomegaly
	c	Cytopenias (affecting ≥2 of 3 lineages in the peripheral blood), hemoglobin <9 g/dl, platelets <100,000 /cm^3^, neutrophils <1,000 /cm^3^
	d	Hypertriglyceridemia and/or hypofibrinogenemia, fasting, triglycerides ≥265 mg/dl, fibrinogen ≤1.5 g/L
	e	Hemophagocytosis in bone marrow or spleen or lymph nodes; no evidence of malignancy
	f	Low or no NK cell activity
	g	Ferritin ≥500 ng/ml
	h	sCD25 (soluble IL-2 receptor) ≥2400 U/ml

Due to its vast differential diagnosis, very few cases of HLH have been reported in our region. In this case, the patient was quite healthy even after two months of illness, and the only symptom was fever. Initially, we were evaluating him for sepsis as a diagnosis. The markedly elevated ferritin levels (10,350 ng/ml) steered us to diagnose HLH. He fulfilled six out of the eight elements of the HLH-2004 criteria, and HScore was 249 showing almost 99% probability of the disease. Regarding the cause, relevant infection screening and malignancy screening as per the affordability of the patient was carried out, but no triggering cause was established.

Treatment of HLH depends upon the triggering cause. Treatment mainly comprises dexamethasone, etoposide, intrathecal methotrexate, and hydrocortisone in patients with central nervous system (CNS) involvement (HLH-94 protocol). Almost half of the patients treated with the above regimen achieve five-year survival [12]. As the prognosis is relatively fair in adults, timely and prompt initiation of HLH protocol is critical. In our case, despite not receiving any treatment, the patient was quite healthy and was able to carry out his routine daily activities. Supportive care is needed for addressing anemia, thrombocytopenia, bleeding, infection, and blood pressure fluctuations. Regular physical examination and monitoring of cell counts, ferritin levels, liver functions, and coagulation profile are important to see the efficacy of treatment. A slower decline rate of serum ferritin after initiation of therapy shows a worse prognosis and is associated with higher mortality [13]. In this case, serum ferritin levels drastically declined after initiating treatment. Allogeneic hematopoietic cell transplant is indicated in HLH gene mutations and if a patient shows no response to initial HLH therapy.

## Conclusions

HLH is often diagnosed late due to lack of awareness of the disease among the physicians and lack of a specific diagnostic test and should be considered in the differential diagnosis of patients with non-resolving fever, hepatosplenomegaly, and cytopenias. Timely evaluation should be done to determine the trigger, which can sometimes be easily treated. As it is a fatal and aggressive disease, timely treatment of HLH and its trigger (especially infections) is crucial.

## References

[1] Filipovich A, McClain K, Grom A (2010). Histiocytic disorders: recent insights into pathophysiology and practical guidelines. Biol Blood Marrow Transplant.

[2] Otrock ZK, Eby CS (2015). Clinical characteristics, prognostic factors, and outcomes of adult patients with hemophagocytic lymphohistiocytosis. Am J Hematol.

[3] Henter JI, Horne A, Aricó M (2007). Diagnostic and therapeutic guidelines for hemophagocytic lymphohistiocytosis. Pediatr Blood Cancer.

[4] Egeler RM, Shapiro R, Loechelt B, Filipovich A (1996). Characteristic immune abnormalities in hemophagocytic lymphohistiocytosis. J Pediatr Hematol Oncol.

[5] Pachlopnik Schmid J, Côte M, Ménager MM (2010). Inherited defects in lymphocyte cytotoxic activity. Immunol Rev.

[6] Aricò M, Danesino C, Pende D, Moretta L (2001). Pathogenesis of haemophagocytic lymphohistiocytosis. Br J Haematol.

[7] Ivashkiv LB, Donlin LT (2014). Regulation of type I interferon responses. Nat Rev Immunol.

[8] Bergsten E, Horne A, Aricó M (2017). Confirmed efficacy of etoposide and dexamethasone in HLH treatment: long-term results of the cooperative HLH-2004 study. Blood.

[9] Allen CE, Yu X, Kozinetz CA, McClain KL (2008). Highly elevated ferritin levels and the diagnosis of hemophagocytic lymphohistiocytosis. Pediatr Blood Cancer.

[10] Palazzi DL, McClain KL, Kaplan SL (2003). Hemophagocytic syndrome in children: an important diagnostic consideration in fever of unknown origin. Clin Infect Dis.

[11] Fardet L, Galicier L, Lambotte O (2014). Development and validation of the HScore, a score for the diagnosis of reactive hemophagocytic syndrome. Arthritis Rheumatol.

[12] Trottestam H, Horne A, Aricò M (2011). Chemoimmunotherapy for hemophagocytic lymphohistiocytosis: long-term results of the HLH-94 treatment protocol. Blood.

[13] Lin TF, Ferlic-Stark LL, Allen CE, Kozinetz CA, McClain KL (2011). Rate of decline of ferritin in patients with hemophagocytic lymphohistiocytosis as a prognostic variable for mortality. Pediatr Blood Cancer.

